# Breeding Strategies and Challenges in the Improvement of Blast Disease Resistance in Finger Millet. A Current Review

**DOI:** 10.3389/fpls.2020.602882

**Published:** 2021-01-08

**Authors:** Wilton Mbinda, Hosea Masaki

**Affiliations:** ^1^Department of Biochemistry and Biotechnology, Pwani University, Kilifi, Kenya; ^2^Pwani University Biosciences Research Centre (PUBReC), Pwani University, Kilifi, Kenya

**Keywords:** allele mining, finger millet, gene pyramiding, *M. oryzae*, marker assisted selection, molecular breeding, QTL mapping, transgenesis

## Abstract

Climate change has significantly altered the biodiversity of crop pests and pathogens, posing a major challenge to sustainable crop production. At the same time, with the increasing global population, there is growing pressure on plant breeders to secure the projected food demand by improving the prevailing yield of major food crops. Finger millet is an important cereal crop in southern Asia and eastern Africa, with excellent nutraceutical properties, long storage period, and a unique ability to grow under arid and semi-arid environmental conditions. Finger millet blast disease caused by the filamentous ascomycetous fungus Magnaporthe oryzae is the most devastating disease affecting the growth and yield of this crop in all its growing regions. The frequent breakdown of blast resistance because of the susceptibility to rapidly evolving virulent genes of the pathogen causes yield instability in all finger millet-growing areas. The deployment of novel and efficient strategies that provide dynamic and durable resistance against many biotypes of the pathogen and across a wide range of agro-ecological zones guarantees future sustainable production of finger millet. Here, we analyze the breeding strategies currently being used for improving resistance to disease and discuss potential future directions toward the development of new blast-resistant finger millet varieties, providing a comprehensive understanding of promising concepts for finger millet breeding. The review also includes empirical examples of how advanced molecular tools have been used in breeding durably blast-resistant cultivars. The techniques highlighted are cost-effective high-throughput methods that strongly reduce the generation cycle and accelerate both breeding and research programs, providing an alternative to conventional breeding methods for rapid introgression of disease resistance genes into favorable, susceptible cultivars. New information and knowledge gathered here will undoubtedly offer new insights into sustainable finger millet disease control and efficient optimization of the crop’s productivity.

## Introduction

Environmental stresses cause reduced growth and significant yield losses in food crops. Avenues for improvement of crops to combat individual and multiple stresses are major breeding goals, and different approaches have been used to improve stress tolerance (Calanca, 2017). Among the biotic stresses, pests and diseases are the most important limiting factors that affect finger millet production worldwide. Finger millet blast caused by the filamentous fungus *Magnaporthe oryzae* (sexual amorph *Pyricularia oryzae*) is the most devastating disease affecting the production and productivity of finger millet because of its destructive nature under favorable conditions and its wide distribution in all finger millet-growing areas (Yoshida et al., 2016). *M. oryzae* infects a finger millet plant during nearly all growth stages and reduces the crop grain yield by up to 100% (Senthil et al., 2012; Takan et al., 2012). Due to the importance of finger millet blast disease, effective disease control measures are needed to ensure global food security, especially in arid and semi-arid regions of African and Asia where the crop is majorly cultivated. Over the years, various pursuits have been made to develop new cultivars that are resistant to the disease. The unavailability of the whole-genome sequence and the limited genomic resources of finger millet has greatly hampered studies of the genetics of resistance to the blast disease compared with other major cereals. As a result of this shortcoming, the understanding of broad-spectrum resistance to finger millet blast disease remains a knowledge gap. Previous work on the pathosystem of the blast fungus on finger millet relied primarily on the phenotypic features and virulence tests using various hosts (Wu et al., 2014; Que et al., 2019; Rasool et al., 2020). These studies concentrated on screening and selection of finger millet cultivars or new advanced lines toward selected strains of blast fungi but with limited success, because the phenotypic traits obtained are extremely variable due to the genetic instability of the blast pathogen. In addition, it often takes a long time and high cost for breeders to develop new varieties with broad-spectrum resistance to blast disease when these strategies are adopted. Additionally, the studies are influenced by environmental pressures and are prone to human errors, leading to ambiguous results.

With the advent of new biotechnological tools, current research strategically focuses on understanding the biotic stresses on finger millet, particularly in advancing the molecular genetics of blast disease in order to develop an integrated management system for blast disease resistance in finger millet. With the advancement of high-throughput sequencing platforms, there has been a tremendous increase in the modern genomic tools available, such as molecular markers, expressed sequence tags (ESTs), gene expression profiling, genome-wide association studies, genetic transformations, and, more recently, genome editing, have been used successfully in various crops to explore the genetic basis of stress tolerance for guidance in the development of plants of superior quality. DNA molecular markers have also been used for population genetics and evaluation of genetic variations that occur between and within plant populations, and their polymorphic structure and level can be invaluable in crop breeding. This review outlines a set of recent molecular and genomic tools that are used to study the finger millet blast fungus.

### Finger Millet and Finger Millet Blast Importance

Finger millet [*Eleusine coracana* (L.) Gaertn.] is an allotetraploid (2n = 4X = 36) member of the Poaceae family. The crop is mostly grown and consumed by people in the poverty-stricken arid and semi-arid tropics of Asia and sub-Saharan Africa (Takan et al., 2012). Its grains have excellent nutraceutical properties, such as high dietary fiber content, amino acids (methionine, phenylalanine, tryptophan, cysteine, isoleucine, and leucine), vitamin B complex, calcium, and iron compared with maize, rice, wheat, and sorghum (Kumar et al., 2016b; Gupta et al., 2017). It is also gluten-free and can be stored for a long period (Chandrasekara and Shahidi, 2010). As a member of the small millets, finger millet is the most climate-resilient crop which can be cultivated under a diverse range of climatic conditions. It ranks fourth on a global scale of production, followed by sorghum, pearl millet, and foxtail millet (Upadhyaya et al., 2007). These qualities make the finger millet an important food and nutritional security crop and a valuable genomic resource. Although the global finger millet production has been increasing, reaching 4.5 million tons in 2018 (FAOSTAT, 2019), the increase is not concomitant with the demand for finger millet because of the rapidly increasing human population and industrialization. To date, no published data exists on associated economic loss due blast disease in finger millet. To overcome this challenge, there is a need to increase finger millet production by at least 40%, like other major cereals (Kumar et al., 2018).

The availability and access to diverse genetic resources is central to genetic improvement of any crop. These genetic resources have to be characterized for their effective utilization in crop improvement programs. Field and *in vitro* genebanks constitute a huge pool of finger millet germplasm collections. As of 2010, 35382 finger millet accessions were conserved in gene-banks across the world. The National Bureau of Plant Genetic Resources (India) and the International Crops Research Institute for the Semi-Arid (ICRISAT) genebanks across the world accounted for 26.9% and 16.8% of the global collections. Other institutions including Kenya Agricultural & Livestock Research Organization (Kenya), National Agricultural Research Organization (Uganda), Ethiopian Biodiversity Institute (Ethiopia), Southern African Development Community (Zambia), and others accounted for the remaining for the remaining 56.3% (FAO, 2010). These finger millet collections are rich in rare alleles for target traits from which researchers and crop breeders can obtain the genetic materials to expedite their work in a sustainable way. However, a systematized usage of genebank accessions has not progressed very far in finger millet research and breeding programs due to the scanty information. The ongoing initiative by ICRISAT and other collaborators to sequence several collections to expedite its use for breeding. It is our view that this effort will provide an opportunity to mine novel alleles for breeding next generation of finger millet varieties.

**FIGURE 1:**
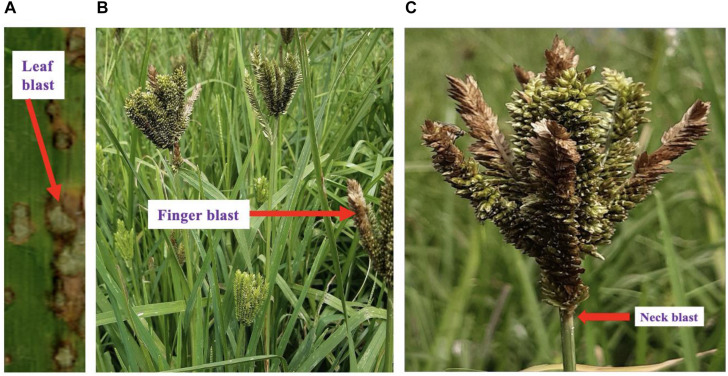
Blast disease symptoms on finger millet **(A)** blast infection of the finger millet l **(B)**. infection on leaves **(C)**. panicle and neck bast symptoms.

Blast disease caused by *M. oryzae* is the most important disease affecting the growth and yield of finger millet. The fungus infects many other economically important crops, such as rice (Mentlak et al., 2012), foxtail millet (Sharma et al., 2014), barley (Tufan et al., 2015), wheat (Cruz and Valent, 2017), and other grass plants within the Poaceae family (Wang et al., 2011; Han et al., 2018). Under favorable conditions, the pathogen infects leaves, stem, collar, node, neck, fingers, and roots, causing substantial crop losses in all finger millet-growing areas. Blast infections occur mostly on the leaves, with the first symptoms of the disease appearing as small gray or brownish dots on the leaves. Following 2–3 days of infection, the dots grow into diamond-shaped lesions of 1.5 cm in length and 0.3–0.5 cm in width, with a white or grayish center (Figure 1). The head blast significantly reduces finger length, seed weight, number of seeds per finger, and total grain yield (Mgonja et al., 2013). The yield losses due to blast have been reported to be between 30 and 50% in large rice-producing areas under favorable environmental conditions (Correa-Victoria and Zeigler, 1993; Skamnioti and Gurr, 2009). Efforts are on-going to develop finger millet varieties with blast resistance. Therefore, continuous studies on blast disease are essential to overcome this disease and thereby sustain finger millet production in the future.

### Biology and Pathogenicity of *M. oryzae*

**FIGURE 2:**
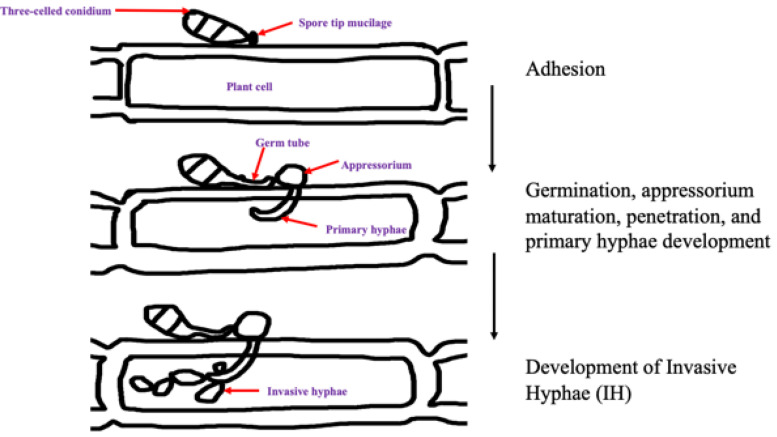
A schematic representation of the infection cycle of *M. oryzae*.

*Magnaporthe oryzae* is plant-pathogenic filamentous ascomycete fungi that belongs to the *Pyricularia* family. Ascomycete fungi are host specific and causes blast disease on more than 50 cultivated and wild monocot plant species (Gladieux et al., 2018). Based on the polyphyletic nature of *Pyricularia* genus, it is believed that reproduction in the finger millet blast fungus is asexual, producing clonal populations (Figure 2). *M. oryzae* is heterothallic and both mating types *MAT1 and MAT2* occur in single mating type gene that has two alleles, *MAT1-1* and *MAT1-2*. However, isolates from single agro-ecological region are usually of only one mating type and where the both mating type idiomorphs occur, the strains cannot interbreed (Takan et al., 2012). Despite this observation, occurrence of highly fertile and hermaphrodite isolates, and isolates haboring the two mating types has been reported (Saleh et al., 2014). Finding suggest that sexual recombination may contribute to genetic variability. Differences have been observed in the two mating types in septoria leaf blotch wheat pathogen *Mycosphaerella graminicola* isolates with *MAT1-1* isolates having significantly greater pathogenicity than *MAT1-2.* On contrary, *M. oryzae* isolates with *MAT1-1* and *MAT1-2* idiomorphs were found to have similar pathogenicity on different monogenetic lines of rice. This has not been tested on finger millet blast strains, and similar results are hypothesized. Despite this suggestion, the finger millet blast fungus sexual recombination, pathogenicity variation, habitat adaptation, and fitness need to be investigated. Further, the phylogenetic evolution and geographic transmission patterns of the finger millet blast pathogen need to be explored.

*Magnaporthe oryzae* infects the host in two stages, the biotrophic stage where it obtains nutrients from live cells and a necrotrophic stage where it obtains nutrients from dead cells (Park et al., 2009). During infection, conidia attach to the host leaf surface by adhesive secretions released from the apical part of the spore tip during hydration. Subsequently, the spore anchors itself tightly to the hydrophobic (non-stick) finger millet surface to allow germination. After that, the conidia produce germ tubes which form a melanized appressoria. A mature appressorium then breaks the leaf cuticle by creating cellular turgor pressure through the accumulation of compatible solutes such as glycerol and secretion of cell wall degrading enzymes; therefore, gaining entry into the epidermal cells (Marcel et al., 2010; Mentlak et al., 2012). Once inside to the host tissues, *M. oryzae* spreads to adjoining cells through the plasmodesmata without causing any perceptible alteration to the cell walls of the host (Galhano and Talbot, 2011). Under favorable conditions of high humidity, the fungus sporulates abundantly from disease lesions, permitting the disease to quickly spread to adjacent finger millet plants and its relatives by wind and water droplets (Mentlak et al., 2012).

### Management Strategies of Finger Millet Blast Disease

Management of blast disease is a challenging issue, and its control relies on three broad strategies various farming practices, application of chemical and biological agents, and breeding of blast resistant varieties. Some of the cultural and farming practices that have been applied to control blast disease include planting time, spacing, crop rotation, nutrient management (nitrogen and silicon), and water management (Mgonja et al., 2013). These farming practices are commonly used by resource-poor subsistence farmers in developing countries who cannot afford other methods of disease management. Preventive and curative chemicals, such as organophosphorus fungicides, critical elements in effective blast disease management not only in finger millet but also in other crops. Treatment of planting seeds with systemic fungicides such as tricyclazole and application of foliar sprays of edifenphos or kitazin with first at the time of ear emergence or application of carbendazim followed by mancozeb after seven to ten days at the same time been demonstrated to be effective in controlling blast disease (Nagaraja et al., 2007). The indiscriminative nature of chemicals often leads to the development of resistance in phytopathogens. Despite their effectiveness, agrochemicals pose potential risks to human health, food safety and the environment. The growing global concern on the environment, coupled with a strong drive to sustainable agriculture, has led to the advancement of non-chemical alternative strategies methods to control blast disease. Biological control (use of microbial antagonists to suppress diseases) of finger millet blast disease has been considered a viable and sustainable alternative method to synthetic chemical fungicides. Bioinoculants offers multiple beneficial aspects such as the production of quality grains, protection of crops against biotic and abiotic stresses; soil fertility enhancement and are environmentally safe. Biological agents have successfully been used to manage fungal diseases, such as powdery mildew (Mgonja et al., 2007; Anand et al., 2010), verticillium wilt disease (Yuan et al., 2017), and anthracnose (Hernandez-Montiel et al., 2018).

Biological control (use of microbial antagonists to suppress diseases) of finger millet blast disease has been considered a viable and sustainable alternative method to synthetic chemical fungicides. Bioinoculants offers multiple beneficial aspects such as the production of quality grains, protection of crops against biotic and abiotic stresses; soil fertility enhancement and are environmentally safe. Biological agents have successfully been used to manage fungal diseases such as powdery mildew (Mgonja et al., 2007; Anand et al., 2010), verticillium wilt disease (Yuan et al., 2017), and anthracnose (Hernandez-Montiel et al., 2018). Attempts have been made to control blast disease using bioinoculants. For example, indigenous rhizosphere *Pseudomonas* sp. strain MSSRFD41, had been shown to control blast disease and promote the growth of finger millet *in vitro* but this approach has not been applied at natural field conditions (Sekar et al., 2018). Similarly, endophytic *Bacillus tequilensis* GYLH001 inhibits the growth of *M. oryzae* rice blast and thus has a high potential application as a bioinoculant for control of rice blast pathogen (Li et al., 2018). These examples offer encouraging results, affirming that bioinoculants can significantly contribute to limit the damage caused by blast disease. However, no effective bioinoculant has been formulated and widely adopted for effective biological control of blast disease pathogen. Effective adoption of bioinoculants requires an improvement in understanding of the complex plant-microbe interactions, and an efficient and stable antagonist for the pathogen under different agroecological conditions must be obtained for this control strategy to be realized. More efforts should also be done to authenticate the currently available outcomes setting up effective formulations and application protocols and deepen the knowledge and awareness on the value of biocontrol agents. Like other blast control strategies, it is our view that biocontrol should not be used alone, but should be implemented in an integrated management framework for sustainable protection of finger millet from *M. oryzae*.

In the current biotechnology era, the breeding of blast-resistant varieties offers the best cost effective and reliable approach for the management of finger millet blast disease, especially in developing countries dominated by subsistence farming. A number of improved finger millet varieties such as such as IE4795, IE 1055, IE 2821, IE 2872, IE 4121, IE 4491, IE 4570, IE 5066, IE 5091, and IE 5537 with broad-spectrum resistance to blast pathogen coupled with desirable agronomic traits such as early flowering, medium stalk length, and high yields have been developed through conventional breeding methods in eastern Africa and Asia. Such The development and deployment in several production systems of these varieties has been a collaborative work of international research institutions and national finger millet breeding programs. The use of such varieties in integrated management of blast disease is desirable because they require minimal fungicides, subsequently lowering production cost. From our unpublished data on appraisal of occurrence, impact, risk factors and farmers’ knowledge and attitudes of finger millet blast disease in Kenya, results showed that many farmers did not know the existence of blast resistant lines and this observation could be the same in all other finger millet growing regions. Farmers should therefore be made aware of the benefits planting resistant lines as opposed to their preferred cultivars. Local finger millet landraces and their wild relatives are commonly used as sources of variation of introgression and hybridization to incorporate the range of useful adaptations for disease resistance into cultivated finger millet. Even though genetic resistance will continue to be the main strategy for control of blast disease, the success of this method is short-lived due to the instability of the *M. oryzae* genome, especially the fast-evolving genes, leading to the breakdown of resistance under field conditions (Mgonja et al., 2013). Abiotic stresses, such as drought, have also displayed a partial or complete breakdown of resistance (Gupta et al., 2017). To overcome this challenge, gene staking/pyramiding and identification of new partial resistance (*R*) genes against finger millet blast disease are an important goal of finger millet breeding. Staking of multiple R-genes or the alleles of a major *R*-gene, which recognize the unique set of *M. oryzae* strains through a conventional breeding approach or transgenesis, has been considered for the attainment of dynamic and durable resistance against different strains of the pathogen (Das et al., 2017; Kumari et al., 2017). Modern biotechnological techniques are simpler, cost-effective, can be performed over a short period, and are more efficient than classical breeding methods.

### Breeding Approaches for Improvement of Disease-Resistant Varieties

The current breeding approaches combine two or more objectives which include increasing grain yield, improving resistance to resistance to various biotic and abiotic stresses and enhancing nutritional quality. Therefore, new finger millet varieties that combine all these traits are desirable. Due to the severity of blast disease, conventional breeding approaches for transferring robust and durable resistance to *M. oryzae* into adapted finger millet germplasm has been a goal of many breeding programs (Upadhyaya et al., 2011; Mgonja et al., 2013). A major challenge for finger millet breeding is that different inheritance models that have been published among the sources of resistance to *M. oryzae* due to the pathogen’s specificity. The drawbacks of conventional breeding due to genetic drag and erosion, reproductive hindrances and longer period it takes has necessitated the need for novel breeding methods. Although some achievements have been achieved through conventional breeding strategies, dynamic, efficient, versatile, and contemporary tools and resources must be continually be developed and applied in order to create the necessary paradigm shift needed in finger millet research and breeding.

The rapid advancement in next-generation sequencing techniques together with the declining associated costs and high-performance computation, have resulted to extensive discovery of numerous genomic resources in plants and other organisms. The wealth of information emanating from the post-genomic era has enabled a better understanding of the physiological, biochemical, and molecular mechanisms involved in genotype and its relationship with the phenotype especially for complex traits, facilitated systematic improvement of crop breeding, and allowed for the efficient use of genetic resources. Novel DNA-driven breeding techniques such as marker-assisted selection (Ghatak et al., 2017), gene pyramiding (Chen et al., 2008; Singh et al., 2013), marker-assisted backcross breeding, (Valarmathi et al., 2019; Ponnuswamy et al., 2020), speed breeding technology (Chiurugwi et al., 2019; Hickey et al., 2019) and a combination of them have been utilized in several crops such as rice, soya bean, maize and wheat. To our knowledge, no finger millet variety has been developed and released based on marker-assisted selection (MAS) technique to date, despite the potential of MAS in other cereal crops improvement has been demonstrated for important traits such as bacterial blight resistance in rice (Pandey et al., 2013). Application of these contemporary approaches will ultimately expedite finger millet breeding efforts against blast disease. However, a lot of knowledge is required before full application of molecular breeding in finger millet as most of the available data for the crop currently is on diversity studies and limited QTLs.

Plant genetic engineering which encompasses genetic transformation and genome editing have opened new avenues to modify crops and provided solutions to solve specific needs, establishing it as one of the most important and dynamic biotechnological tools to revolutionize agriculture. This technology can integrate foreign genetic material into different plant cells to produce transgenic plants with new desirable traits, such as drought tolerance, pest and disease resistance, and quality improvement. To circumvent the controversy of genetically modified organisms, innovation in genome editing tools that cause genome changes without producing transgenic plants are currently being explored (Luo et al., 2015; Miroshnichenko et al., 2019).

As an orphaned crop, the status of finger millet genetics and genomics still lags behind that of 204 other food cereal crops, such as maize, rice, wheat, and barley, and even other small millets due to limited research interests and investments. Few biotechnological approaches have been tested on finger millet for crop improvement (Sood et al., 2019). However, this reality is rapidly evolving as the cost of technologies decreases, leading to an exponential decline in the cost of the generation of new knowledge. A vast reservoir of more than 28,041 finger millet germplasm is available in various institutions worldwide for genetic and breeding research (Ceasar et al., 2018). The long-awaited whole-genome sequence and annotation will trigger much higher-resolution research on functional genomics, proteomics, comparative genomics and forward and reverse genetics to unravel the molecular mechanisms mediating major agronomic traits, such as yield, grain quality, abiotic stress tolerance, and pest and disease resistance. Subsequently, the genomic knowledge will be transferred into crop productivity through molecular breeding and better agronomic husbandry.

With the expected large amounts of omics sequencing data, finger millet scientists need to prepare the emerging opportunities and challenges for multi-omics big data integration by means of artificial intelligence for a feasible improvement approach. Studies on genome collinearity show a high genomic synteny between finger millet and rice, foxtail millet and maize, in that order (Hittalmani et al., 2017; Pandian and Ramesh, 2019). Blast resistance in finger millet has been studied using comparative genomics, and different approaches have been used to genetically improve finger millet for effective, durable resistance to important diseases. Several *R*-genes and quantitative trait loci (QTL) in finger millet linked to the blast pathogen have been reported and sequenced (Ramakrishnan et al., 2016; Odeny et al., 2020). These genes and QTL show high sequence similarity in rice and barley, signifying a common evolutionary ancestry for these *R*-genes (Ramakrishnan et al., 2016). Similar methods could be used to identify novel alleles for blast resistance through syntenic studies with the data available from rice, pearl millet, barley, and other related plants. To date, there is no literature reporting mutation breeding in finger mullet to generate new varieties. Still, this avenue can be explored given the successful mutation breeding of rice, which has focused mainly on grain quality and taste, agronomic traits, and resistance against pests and diseases. Traditional finger millet landraces have been widely used as genetic resources of breeding programs (Mirza and Marla, 2019). However, the lengthy 10–15-year breeding cycle from crossing to variety release slows the progress.

The majority of the traits, such as resistance to blast disease, are polygenic, and this poses a major challenge when combining large numbers of traits. To supplement conventional breeding, shorten breeding cycles, and accelerate research activities, powerful tools, such as speed breeding protocols, which accelerate plant growth and development, may be explored in finger millet breeding strategies (Watson et al., 2018). Speed breeding technology is able to achieve up to six generations per year of barley (Hickey et al., 2017), wheat (Watson et al., 2018), and oats (González-Barrios et al., 2020), presenting a robust tool to reduce the long period of breeding cycles effectively. Several speed breeding protocols that utilize prolonged photoperiods and controlled temperatures to accelerate growth and development have been developed for the world’s major cereals. Finger millet is a tropical, short-day plant, but its speed breeding protocol is yet to be developed. In order to realize the actual and potential opportunities of speed breeding technology, it is essential to optimize the parameters at a low cost for finger millet. Being time and resource saving, speed breeding technology will accelerate research, improve stability, and increase global finger millet production to meet food security demands of the increasing population.

### Blast Disease Improvement Due to Markers and Genotyping Systems

Molecular markers are highly treasured in plant genetics. Over the years, molecular markers have played a prominent and versatile role in finger millet breeding for cultivar improvement, taxonomy, population genetics, plant physiology, and genetic engineering (Veluru et al., 2020). Characterization of finger millet using isozyme makers found fixed heterozygosity at several loci that was identical across the examined accessions (Werth et al., 1994). DNA-based makers such as restriction fragment length polymorphism (RFLP), amplified fragment length polymorphism (AFLP), simple sequence repeat (SSR), expressed-sequenced tag (EST), and markers have been used to generate genetic map of finger millet (Dida et al., 2007; Babu et al., 2014a). Both isozyme and DNA marker analyses have indicated a low variation within cultivated finger millet. Highly variable makers will therefore be required for their application in the crop’s breeding.

The advancement in high-throughput sequencing techniques has facilitated sequencing for whole genome sequencing and re-sequencing projects, generating large volumes of sequence data quickly and at a reasonable cost. Next generation sequencing techniques presents new avenues for high-marker density genotyping procedures such as genotyping by sequencing (GBS), which can be used to unravel large numbers of single nucleotide polymorphisms (SNPs) for species identification, diversity analysis, linkage mapping, and genome-wide association studies (GWAS) (Elshire et al., 2011). Because of its high throughput and robustness, GBS can be used to unravel the close variation of cultivated finger millet genotypes. GBS was successfully applied to establish the genetic diversity, population structure and ploidy level among 112 *Vanilla planifolia* accessions and identified 521,732 SNP markers (Hu et al., 2019). Further, genetic diversity of olive germplasm (*Olea europaea* L.) was achieved through GBS technology (Zhu et al., 2019). These results validate the efficacy of genomics-based tools in species genotyping and demonstrate GBS as an effective marker for cultivar genetic diversity analysis in several cultivated crops, providing a vital tool for genomics-assisted plant breeding. Although the genome sequence for finger millet has not been release so far, considerable gains of GBS have been achieved. For example, Tiwari et al. (2020) discovered genes and QTLs governing seed protein content and related traits in finger millet using SNPs discovered via GBS technology. Likewise, GBS was used to identify genomic regions which govern grain nutritional traits in finger millet and generated 169,365 SNPs and three subpopulations (Puranik et al., 2020). These two examples signify the utility of GBS in genome wide association analysis in mining of novel fundamental genetic information which is essential for marker-assisted breeding against blast disease. The limitations which may arise is that many of the SNPs identified from blast infection are likely to be associated with broad stress or infection responses.

The main challenge posed by the use molecular markers in plant breeding is the high cost of establishing, maintaining of molecular laboratories and inadequate qualified human resource. Moreover, the huge capital requirement for development of markers another major impediment in use of molecular markers in plant breeding programs especially in many developing countries where finger millet is grown. These countries should put more efforts to surmount these challenges. These challenges could be resolved through establishment of specialty regional and continental molecular laboratories could cut costs and bring synergy in research and plant breeding activities.

### Resistant Genes and QTLs for Blast Disease

Molecular marker-based breeding approaches have been valuable in the development of blast resistance and in improving important agronomic traits in crops, such as rice and foxtail millet (Tabien et al., 2002). The majority of these traits, for instance, blast resistance, are under quantitative genetic control (Fu, 2015). Nine blast *R*-genes (*Pita*, *Pi9*, *Pi2*, *Piz-t*, *Pi-kh*, *Pi36*, and *Pi37*) belonging to the nucleotide-binding site–leucine-rich repeat (NBS-LRR) family have been cloned in rice by using different strategies (Ameline-Torregrosa et al., 2008). EST sequences of NBS-LRR regions of finger millet have shown homology with *Pi-kh* and *Pi21*, indicating that rice blast *R*-gene orthologs may be playing a crucial role in conferring resistance in finger millet (Kumar et al., 2016a). Genetic mapping and molecular characterization of quantitative traits enable genome-aided breeding in improving the finger millet crop. The common tools used for analyzing the quantitative traits are association mapping and linkage analysis. Association mapping for resistance has been done using genic-SSR markers strongly linked to blast QTL from the finger millet NBS-LRR region in the identification of QTL for finger blast and neck blast resistance. Babu et al. (2014b) identified five significant QTL for finger blast and neck blast. The finger blast QTL were strongly associated with the genic-SSR primer FMBLEST32 and rice SSR RM262 (Babu et al., 2014a). The FMBLEST32 marker was designed from a *Pi5* rice blast gene known for a relatively broader spectrum resistance to *M. oryzae* (Wang et al., 1994). Ramakrishnan et al. (2016) identified two leaf blast resistance QTL strongly associated with markers UGEP101 and UGEP95 by association mapping.

### Gene Pyramiding for Blast Resistance

The notable losses of finger millet to blast disease necessitates the development of highly improved and novel strategies to enhance the capacity of various finger millet varieties that can survive attacks caused by the ever-evolving, mutating *M. oryzae* pathogen while also enduring the variable farming and climatic conditions with a high level of grain quality. Although fungicides are an option for the control of blast disease, they are expensive or not readily available to subsistence and smallholder farmers who are the dominant producers of finger millet in the tropics of sub-Saharan Africa and Asia. Finger millet varieties with resistance to *M. oryzae* fungi offer a cost-effective, easy-to-use, and environmentally-friendly management strategy (Pastor-Corrales et al., 1998). Several sources of blast disease resistance have been identified among primary and secondary gene pools of finger millet (Wang et al., 1994; Babu et al., 2014b; Ramakrishnan et al., 2016; Odeny et al., 2020), However, development of varieties with durable blast disease resistance is difficult. In particular, the broad and dynamic virulence diversity of the blast pathogen has been a limiting factor for host-plant resistance to blast disease because it renders the resistant varieties susceptible within a short period (Odeny et al., 2020).

The advancement of molecular methods in plant breeding has significantly broadened the identification of various *R*-genes in finger millet and other important crops of the Poaceae family. The presence of a set of different *R*-genes in the same plant averts the infection from multiple pathogen races, thereby avoiding fungal evolution by preventing recombination between different fungal races. Previous evidence on rice blast disease has shown that integrating a set of different valuable *R*-genes or QTLs into the same plant would block the infection from several pathogen strains, consequently preventing the fungal evolution through the averting recombination between different fungal races (Yasuda et al., 2015). Gene pyramiding involves stacking of multiple-genes, which results in the simultaneous expression of the various genes in the same plant (Delmotte et al., 2016). Although ahis approach looks promising in compacting blast and other fungal disease in rice and other cereal crops such as maize (Zhu et al., 2018) and wheat (Cruppe et al., 2020), although, it has not been tried in finger millet. An assessment of the performances of three approaches to control root-knot nematode: cultivar mixtures, crop rotation, and pyramiding of *R-*genes in pepper and lettuce under controlled conditions for more than 3 years (Djian-Caporalino et al., 2014). Results from their work demonstrated that pyramiding of different genes conferring resistance to root-knot nematodes in one genotype was more durable and suppressed the emergence of virulent isolates than pyramiding of different genes conferring resistance to root-knot nematodes in cultivar mixtures and crop rotations. These empirical results, together with theoretical considerations of qualitative and quantitative disease resistance and retrospective analysis, pinpoint that gene pyramiding is the most powerful strategy to provide durable resistance to plant pathogens.

However, gene pyramiding has challenges, including compromised efficacy of stacking genes, if critical assumptions are not adhered to and virulence gene mutations occur, which are often independent of one another. Moreover, the masking of gene expression of resistance, genotype × environment interactions, the phenotypes, and physiological and biochemical penalties linked with *R*-genes, could eviscerate the agronomic performances. The long period required to obtain a successful variety through gene pyramiding is another major impediment, especially to seed companies. This challenge has been resolved by the new approaches, such as novel sequencing technologies, marker-assisted selection, genetic engineering, and genomic editing. These new methods have aided in the discovery of new, essential *R-*genes with ease and facilitated their combination into a single variety. The breakdown of pyramided genes has also been recorded in several experiments and explained the theoretical projection (Delmotte et al., 2016; Rana et al., 2019). In turn, this creates probabilities of the emergence of multi-virulent pathogen strains, such as *M. oryzae*. Therefore, it is imperative to strike a balance between an economic impact and effective strategies for controlling the disease.

### Allele Mining and Blast Resistance Genes

The advancement made in breeding of superior crops has been achieved by to gathering of valuable alleles from vast plant genetic resources from different agro-ecological regions of the world. The wild relatives and landraces of crops still have numerous untapped valuable alleles which could be sustainably exploited for development of superior cultivars which are able withstand environmental variations and still retain the preferred qualities. Introgression of novel alleles from wild relatives into cultivated crop varieties such as stripe rust resistant wheat (Liu et al., 2020), tomato against tomato leaf curl virus, late blight and root knot nematodes (Kumar et al., 2019), development of a rice mega rice variety “Tellahamsa” for bacterial blight and blast resistance (Jamaloddin et al., 2020) as proven that specific alleles and their combinations produce dramatic trait changes when introgression into a suitable genetic background. At the moment, no report exists on gene pyramiding on finger millet. More efforts should therefore be done to unravel more new important alleles to continually enrich the genetic potential of crops. The techniques and prospects of allele mining in the genomic era has been extensively reviewed (Kumari, 2018), so it is not discussed in detail here.

Together with other constraints, blast disease causes a yield loss of as high as 100% in areas infested with the pathogen (Mgonja et al., 2013), purporting a need to understand the molecular mechanism of blast resistance and identify *R*-genes for the blast disease. With the advancement of sequencing technology, enormous sequence and expression data has been deposited into various databases. The use of these novel genomic tools has accelerated discovery and annotation of novel genes and further facilitated the development of allele-specific-markers. Due to the scarcity of genomic resources for genetic improvement of finger millets, comparative genomics will play a critical role in analyzing the most useful and essential agronomic traits, like blast resistance (Kumar et al., 2016a). Comparative analysis of finger millet and rice genomes has demonstrated that most of the chromosomes are highly collinear with 85% synteny (Srinivasachary et al., 2007). Synteny relationship between rice and rice mapped blast *R*-genes through association mapping using NBS-LRR EST sequences, *M. grisea* and *Pi* rice genes of rice. Babu et al. (2014b) found that the finger millet blast and neck blast QTL were linked to rice genes, such as *Pi5*, *Pi21*, *Pi-d*(*t*), and NBS-LRR. Therefore, these rice blast *R*-genes (*Pi5*, *Pi21*, *Pi-d*(*t*), and NBS-LRR) can be targeted for allele mining in finger millet.

### Transgenesis for Blast Resistance

The improvement of finger millet using biotechnological tools has lagged when compared with the research made in other major cereals. Genetic engineering of finger millets is essential to improve the nutritional quality and resistance to abiotic and biotic stresses. Improvement of crops through biotechnological techniques depends largely on successful and efficient plant tissue culture protocols that can be categorized into direct organogenesis, indirect organogenesis, and somatic embryogenesis (Loyola-Vargas and Ochoa-Alejo, 2018). Previous studies on finger millet have identified several inherent challenges associated with *in vitro* regeneration, such as the severe recalcitrant nature, polyploidy, and genotypic dependence, which singly or collectively frustrate the plant tissue culture work and, consecutively, the crop improvement systems through transgenesis (Dosad and Chawla, 2016). Plant regeneration in finger millet using different explants in different genotypes has been reported, such as epicotyl (Patil et al., 2009), shoot apical meristem (Babu et al., 2018; Ngetich et al., 2018), mature seeds (Bayer et al., 2014; Pande et al., 2015), and mature embryos (Satish et al., 2016). These protocols provide an opportunity to improve *in vitro* plant regeneration studies in finger millet, although optimization is required for each genotype. To date, there is no literature on *in vitro* regeneration of finger millet using anther culture, protoplast, and protoplasmic fusion. Attempts should also be made to establish a genotype-independent *in vitro* regeneration system for finger millet.

Various protocols have been used for genetic engineering of finger millet, including biolistic (Gupta et al., 2001), microprojectile bombardment (Latha et al., 2005), and *Agrobacterium tumefaciens*-mediated transformation (Ceasar and Ignacimuthu, 2011; Hema et al., 2014). Among them, *Agrobacterium-*mediated transformation is the most successful and frequently used method to deliver DNA for the production of transgenic finger millet. However, there have been few attempts to use these procedures for developing transgenic finger millet lines resistant to blast disease. Latha et al. (2005) produced transgenic finger millet plants resistant to leaf blast disease by using the biolistic transformation technique to introduce a gene coding for an antimicrobial peptide of prawn. In other work, finger millet plants conferring resistance to leaf blast disease were developed via *Agrobacterium*-mediated genetic transformation of a rice chitinase (*chi11*) gene (Ignacimuthu and Ceasar, 2012). Results from these two studies demonstrated a high level of resistance to leaf blast disease in the transgenic plants compared with the untransformed control plants. Blast disease also affects the neck and the fingers, yet there is no reported work on transgenic finger millet resistant to these crop organs. A transgenic approach for developing finger millet with broad and durable blast resistance necessitates screening many potential antifungal genes and pyramiding of possible genes because the fungus is highly variable and can often overcome the deployed blast-resistant cultivars in a short period when resistance is dependent on one major *R*-gene. Stacking multiple *R-*genes into a single variety through genetic transformation is a promising tool for breeding durable and superior resistance, especially when the *R*-genes emanate from different gene clusters and different host resistance interactions between the *R*-genes and their effector proteins are provided (Dong and Ronald, 2019). The whole-genome sequence of finger millet, which is anxiously being awaited by finger millet research enthusiasts, is expected to be exploited to facilitate the new genome editing tools, especially, the CRISPR/Cas system. CRISPR (clustered regulatory interspaced short palindromic repeats) and its associated proteins (Cas) guide the complex to cleave complementary DNA. The CRISPR/Cas system is revolutionary and innovative tool for plant genome editing because of its simplicity, a wide range of applications, and is cost-effective (Langner et al., 2018). In order to produce only genome edited plants lacking any foreign DNA inserts. It is therefore vital to establish a protoplast-based regeneration system for finger millet to accomplish the goal of producing blast-resistant finger millet and also propel research and innovation in the crop to the next and higher level comparable to rice, wheat, barley, and maize.

### Integrating Disease Resistance Genes With Other Crop Disease Control Strategies

In a permanently dynamic world and society, environmental protection and agricultural sustainability remain the core drivers for food security. Despite the technological advancements made over the past two decades, a real food crisis due to plant diseases has emerged as a significant threat to food security worldwide. Integration of durable disease *R*-genes in the advent of pathogen evolution caused by climate change perturbations and other evolutionary pressures provides sturdy protection against crop diseases. It complements a diversified, integrated management of *M. oryzae* because the simultaneous use of m control ‘weapons’ guarantees maximum returns. Empirical studies and theoretical models demonstrate that an amalgamation of different selective pressures delays the emergence of virulence (Anderson et al., 2019). For example, the durability of an introgressed *R*-gene(s) targeting a pathogenic fungus, such as *M. oryzae*, could be significantly elevated through the application of fungicides targeting that particular pathogen. Generally, all agricultural practices intended to control a given pathogen should theoretically be integrated to increase their respective effectiveness and durability (Anderson et al., 2019; Hu et al., 2020). In cognizance of smallholder farmers who predominantly cultivate finger millet, such combinations may be constrained by financial, technical knowhow, human health, and environmental factors. To achieve the goal of integrated management of blast disease in finger millet, community engagement and extension services, a healthy partnership between all players in the finger millet value chain and training programs should be emphasized to achieve long-term success.

### Conclusion

Effective disease management strategies are crucial to sustaining the production of high-quality crops, as well as reducing the environmental impacts attributable to pathogens and their management measures. *M. oryzae* is the causative agent of blast, the most damaging disease of finger millet, affecting the finger millet production and causing massive yield loss of up to 80% of finger millet yield per annum globally (Mgonja et al., 2013). Information on the genetic identity of *M. oryzae*, as well as its pathogenesis, is important for the precise development of finger millet varieties with different *R*-genes. The development of resistant varieties with durable resistance through the introgression of new genes into a variety is an effective, economical, environmentally friendly, and sustainable approach to controlling the finger millet blast disease. Various molecular tools are available that facilitate the mining of many genes of interest, such as blast *R*-genes and QTL, and identification of their sources with ease, which presents an opportunity for efficient improvement of finger millet through different breeding techniques. Gene pyramiding, allele mining, speed breeding, genetic engineering, genome editing, and other novel molecular breeding approaches present possibilities to attain durable resistance against the bast disease pathogen in finger millet. Blast disease control by combining disease *R*-genes with other methods could be expected to improve the durability of genetic resistance in improved finger millet cultivars.

## Author Contributions

WM conceptualized the manuscript and critically edited the manuscript for publication. WM and HM undertook the literature review, analysis, and wrote the manuscript. Both authors contributed to the article and approved the submitted version.

## Conflict of Interest

The authors declare that the research was conducted in the absence of any commercial or financial relationships that could be construed as a potential conflict of interest.
